# Type-4 Resistant Starch in Substitution for Available Carbohydrate Reduces Postprandial Glycemic Response and Hunger in Acute, Randomized, Double-Blind, Controlled Study

**DOI:** 10.3390/nu10020129

**Published:** 2018-01-26

**Authors:** Maria L. Stewart, Meredith L. Wilcox, Marjorie Bell, Mary A. Buggia, Kevin C. Maki

**Affiliations:** 1 Global Nutrition R & D, Ingredion Incorporated, 10 Finderne Ave, Bridgewater, NJ 08807, USA; 2 Midwest Biomedical Research, Center for Metabolic and Cardiovascular Health, 489 Taft Ave Suite 202, Glen Ellyn, IL 60137, USA; mwilcox@mbclinicalresearch.com (M.L.W.); mbell@mbclinicalresearch.com (M.B.); mbuggia@mbclinicalresearch.com (M.A.B.); kmaki@mbclinicalresearch.com (K.C.M.)

**Keywords:** resistant starch type 4, dietary fiber, post-prandial, blood glucose, insulin, capillary glucose, venous glucose, glycemic response, satiety, gastrointestinal tolerance

## Abstract

Resistant starch (RS) is a type of dietary fiber that has been acknowledged for multiple physiological benefits. Resistant starch type 4 (RS4) is a subcategory of RS that has been more intensively studied as new types of RS4 emerge in the food supply. The primary aim of this randomized, double-blind, controlled study was to characterize the postprandial glucose response in healthy adults after consuming a high fiber scone containing a novel RS4 or a low fiber control scone without RS4. Secondary aims included assessment of postprandial insulin response, postprandial satiety, and gastrointestinal tolerance. The fiber scone significantly reduced postprandial glucose and insulin incremental areas under the curves (43–45% reduction, 35–40% reduction, respectively) and postprandial glucose and insulin maximum concentrations (8–10% and 22% reduction, respectively). The fiber scone significantly reduced hunger and desire to eat during the 180 min following consumption and yielded no gastrointestinal side effects compared with the control scone. The results from this study demonstrate that a ready-to-eat baked-good, such as a scone, can be formulated with RS4 replacing refined wheat flour to yield statistically significant and clinically meaningful reductions in blood glucose and insulin excursions. This is the first study to report increased satiety after short-term RS4 intake, which warrants further investigation in long-term feeding studies.

## 1. Introduction

Dietary fiber encompasses a wide range of non-digestible carbohydrates with multiple physiological benefits, and it is noted as a short-fall nutrient in Western diets [1,2]. One such physiological benefit is improved blood glucose control. Postprandial blood glucose management has been well-documented among viscous fibers, such as oat beta-glucan, due to attenuated glucose absorption in the small intestine [3]. Decreased postprandial blood glucose is also observed when fibers, such as resistant starch (RS), replace available carbohydrate in food formulations [4]. Postprandial blood glucose control has long been recognized as a predictor of diabetes development. More recently, poor postprandial blood glucose control correlated with the presence of coronary heart disease [5], thus demonstrating the value of improved postprandial blood glucose control.

As noted previously, RS can reduce postprandial blood glucose, particularly when replacing refined wheat flour in product formulations [4,6]. The majority of clinical research on RS evaluated the effects of resistant starch type-2, which is a granular, native starch, resistant to digestion, and resistant starch type-3, which is a retrograded starch that resists digestion. Fewer clinical studies have been conducted on resistant starch type-4 (RS4, chemically modified starch that resists digestion by intestinal enzymes). The category of RS4 is diverse, with a range of starch bases and chemical modifications existing in the food supply. These attributes can affect functionality in a food product, and digestibility and fermentability after consumption [7]. Due to these differences, it is critical to evaluate food applications and physiological effects of each, specific type of RS4. Within the category of RS4, the most studied type is phosphated distarch phosphate [8,9,10,11,12,13]. Fewer studies have been conducted on distarch phosphate [14,15,16], hydroxypropyl distarch phosphate [17,18] and only one study has been conducted on RS4 that is acid hydrolyzed and heat treated, to date [19].

Among the aforementioned studies, the most frequent, primary outcome was postprandial blood glucose. Additional outcomes of the acute studies included satiety, energy expenditure, and substrate utilization. Long-term studies of RS4 examined its effects on blood glucose, blood lipids, and gut microbiota. The primary aim of the present study was to characterize the postprandial blood glucose response in healthy adults to a novel RS4 (acid hydrolyzed and heat treated maize-based RS) in a ready-to-eat baked good. Secondary aims were to evaluate postprandial insulin response, satiety, and gastrointestinal tolerance. We hypothesized that replacement of digestible carbohydrate from refined wheat flour with RS4 would reduce postprandial blood glucose.

## 2. Materials and Methods

### 2.1. Study Design and Study Visits

This double-blind, randomized, controlled study was conducted in accordance with the Declaration of Helsinki (2000). The protocol was reviewed and received ethical approval before initiation of the trial (Aspire IRB, Santee, CA, USA; protocol number MB-1702). All subjects provided informed, written consent before any study procedures were conducted. The subjects attended 3 study visits (visit 1, day 7, screening; visit 2 day 0, treatment 1; visit 3, day 7, treatment 2). Female subjects of child bearing ability attended study visits during the follicular phase of their menstrual cycles. On visit 2, subjects were randomly assigned to one of two treatment sequences. Prior to visit 2, subjects completed a 24-h diet record. Subjects arrived to the study center fasted on the morning of visits 2 and 3. Baseline satiety was assessed 30 min prior to study product consumption, and baseline blood samples were taken 15 min prior to study product consumption. Subjects consumed the study scone and 240 mL of water within 10 min. Subjects were offered an additional 178 mL of water after they consumed the study scone. Blood measurements and satiety assessment were conducted during the 180 min following the onset of scone consumption. Prior to visit 3, the subjects were instructed to replicate their diet, based on the 24-h diet record from visit 2. Further details are provided in Section 2.4.

### 2.2. Study Subjects

Subjects were recruited from the Boca Raton area in Florida, USA. Subjects were enrolled in the study after meeting inclusion (age 18–74 years, body mass index 18.5–29.99 kg/m^2^, general good health, women of child bearing potential had to be willing to commit to a medically approved form of contraception for the duration of the study) and exclusion criteria (fasting capillary glucose ≥ 5.55 mmol/L, major trauma or surgical event within 3 months of screening, current or recent history of drug or alcohol abuse, ≥4.5 kg weight change in past 2 months prior to screening, uncontrolled hypertension, recent use of antibiotics or signs or symptoms of active infection, extreme dietary habits, recent consumption of foods fortified with and/or containing supplements containing probiotics or who had used medications known to influence carbohydrate metabolism, gastrointestinal motility, satiety, appetite, taste, sense of smell, or weight, women only who are currently pregnant or lactating). Subjects agreed to certain study restrictions such as having no plans to change smoking habits or other nicotine use during the study, abstaining from use of tobacco products during study visits, willingness to maintain body weight and habitual diet throughout trial, attempting to replicate the pre-visit 2 diet during the 24 h pre-testing period before visit 3, abstaining from alcohol consumption, and abstaining from vigorous physical activity for 24 h prior and during test visits.

### 2.3. Study Foods

The fiber scone contained VERSAFIBE™ 2470 resistant starch (Ingredion Incorporated, Bridgewater, NJ, USA), which was the primary source of fiber in the scone. VERSAFIBE™ 2470 resistant starch is a resistant starch type 4 with 70% dietary fiber (AOAC 2009.01). VERSAFIBE™ 2470 resistant starch is produced from food grade high-amylose maize starch. Digestibility of the high-amylose maize starch is decreased through acid hydrolysis and heat treatment, which results in increased RS4 and total dietary fiber (TDF) in the finished product, VERSAFIBE 2470 resistant starch. Because the ingredient is produced from high-amylose maize starch, the nondigestible carbohydrate (fiber) in VERSAFIBE 2470 resistant starch is RS4. There are no nonstarch polysaccharides in the ingredient.

The fiber scone and control scone were matched for weight, fat, protein, sugar and total carbohydrate (Table 1). The portion size was selected based on typical scones in the United States marketplace. Nutrient composition of the scones was analyzed by Medallion Labs (Minneapolis, MN, USA) using standard methods of analysis (fats AOAC 996.06, protein AOAC 992.15, sugar AOAC 977.20, and dietary fiber AOAC 2009.01). Total carbohydrate was determined through calculation by difference. Energy content was calculated using Atwater factors (9 kcal/g fat, 4 kcal/d protein, 4 kcal/g available carbohydrate). Control scone and fiber scone formulations are available in Supplementary Table S1. The fiber and control scones were identical in appearance. The scones were packaged in identical opaque envelopes with a numeric code for identification. Neither the study subjects nor the investigators knew the identity of the scones. The subjects rated the scones on palatability after consumption.

### 2.4. Measurements

#### 2.4.1. Capillary Glucose

The TRUEtrack™ Blood Glucose Monitoring System and the TRUE METRIX^®^ Blood Glucose Meter (Trividia Health Inc., Fort Lauderdale, FL, USA) were used for determination of capillary glucose at screening (visit 1, day 7) and at test visits 2 and 3 (days 0 and 7). At screening, a single fasting glucose test was performed, and at test visits, the capillary glucose was measured at *t* = −15 ± 2 min, 15, 30, 45, 60, 90, 120 and 180 min ± 2 min, where *t* = 0 was the start of study product consumption. The test-retest % CV for tests on different days was similar for the fasting capillary (9.0%) and venous (10.4%) samples.

#### 2.4.2. Venous Glucose and Insulin

Venous samples were drawn from an indwelling catheter that was placed at least 10 min prior to the first sample collected. The catheter was flushed regularly with normal saline to maintain patency. At visits 2 and 3 (days 0 and 7), venous samples for measurement of plasma glucose and insulin concentrations were collected at *t* = −15 min ± 2 min and at *t* = 15, 30, 45, 60, 90, 120 and 180 min ± 2 min, where *t* = 0 min was the start of study product consumption. The Cleveland Heart Lab (Cleveland, OH, USA) conducted the plasma glucose and insulin analyses. Glucose was measured using a hexokinase/glucose-6-phosphate dehydrogenase enzymatic assay on an automated assay instrument (Roche Cobas Mira Plus Chemistry System, Roche Diagnostic Systems, Indianapolis, IN, USA), and insulin was measured with an electrochemiluminescence immunoassay (Cobas e 411 Immunoassay Analyzer, Roche Diagnostics, Indianapolis, IN, USA) [20].

#### 2.4.3. Satiety Visual Analog Scales (VAS)

At visits 2 and 3 (days 0 and 7), satiety VAS ratings were assessed for fullness, hunger, desire to eat, and prospective consumption at *t* = −30, 30, 60, 90, 120, 150, and 180 min ± 2 min, where *t* = 0 min was the start of study product consumption. The questions were “How hungry do you feel?” with anchors at 0 and 100 mm of “Not hungry at all” and “As hungry as I’ve ever felt”; “How full do you feel?” with anchors of “Not full at all” and “As full as I’ve ever felt”; “How strong is your desire to eat?” with anchors of “Not at all strong” and “As strong as I’ve ever felt”; “How much food do you think you can eat?” with anchors of “Nothing at all” and “A large amount.”

#### 2.4.4. Gastrointestinal (GI) Tolerability Questionnaire

A GI Tolerability Questionnaire [21] was administered at visits 2 and 3 (days 0 and 7) to assess the presence and severity of selected GI symptoms including nausea, GI rumblings, abdominal pain, bloating, flatulence, and diarrhea during the 0–180 min time period, where *t* = 0 min was the start of study product consumption. GI symptoms were scored as follows: 0 = none, 1 = no more than usual, 2 = somewhat more than usual, and 3 = much more than usual.

#### 2.4.5. Palatability

At the end of visits 2 and 3 (days 0 and 7), subjects completed a study product palatability questionnaire [22,23] in which they rated the study products on a scale from 1 (dislike extremely) to 10 (like extremely) on appearance, texture, flavor, and overall acceptance of the scones; on a scale from 1 (not at all healthy) to 10 (extremely healthy) on how healthy they thought the scone was; and on a scale from 1 (very little) to 10 (very nutritious) on how nutritious they thought the scone was. Subjects also responded to statements of “I recommend the scone to family and friends” and “I tolerated the muffin well, with no complaints” by marking rating of strongly disagree (−2), disagree (−1), no opinion (0), agree (1), or strongly agree (2).

### 2.5. Data Analysis

#### 2.5.1. Outcome Variables

The primary outcome variable was the difference between treatment conditions in the incremental area under the curve (iAUC) for venous glucose from 0 to 120 min. The secondary outcome variables were the differences between treatments in the following parameters: venous glucose iAUC from 0 to 180 min; capillary glucose iAUC from 0 to 120 min and from 0 to 180 min; insulin iAUC from 0 to 120 min and from 0 to 180 min; maximal concentrations (Cmax) for glucose (venous and capillary) and insulin (venous); and the hunger, fullness, desire to eat and prospective consumption net incremental area under the curve (niAUC) from 0 to 120 min. For the iAUC calculation of the venous and capillary glucose and venous insulin measures, the pre-consumption measurement at *t* = −15 min was counted as *t* = 0. For the niAUC calculation of the satiety VAS measures, the pre-consumption measurement at *t* = −30 min was counted as *t* = 0. Areas under the curve were calculated using the trapezoidal rule [24].

#### 2.5.2. Sample Size

An evaluable sample of 29 subjects was expected to provide 80% power to detect a difference of 10% in the primary outcome variable of venous glucose iAUC_0–120 min_ between treatment conditions. This calculation was based on a paired *t*-test with an expected standard deviation of 18.5% based on previous studies conducted by the investigators. No adjustment to the alpha level was planned for comparisons between treatments for secondary outcome variables. A total of 36 subjects were randomized allowing for anticipated subject attrition.

#### 2.5.3. Statistical Analyses

All tests of significance were assessed at alpha = 0.05, 2-sided. Statistical analyses were conducted using SAS for Windows (version 9.3, Cary, NC, USA). Missing data were not imputed and only observed data were included in the statistical models. Descriptive statistics (number of subjects, mean, standard error of the mean (SEM), standard deviation, median, interquartile limits, minimum and maximum for continuous variables; counts and percentages for categorical variables) were calculated by study product. Baseline comparability of sequence groups was assessed by analysis of variance (ANOVA) with treatment sequence as a fixed effect for continuous variables. For categorical variables, baseline comparability was assessed by chi-square test.

For analyses of the continuous outcome variables and continuous palatability scores, differences in responses between study products were assessed using SAS Proc Mixed repeated measures ANOVA. Initial repeated measures ANOVA models contained terms for treatment, sequence, and period with subject as a random effect. Models were reduced in a stepwise manner until only significant (*p* < 0.05) terms or treatment remained in the model. Assumptions of normality of residuals were investigated for each response measurement. In cases when the normality assumption was rejected at the 1% level with the Shapiro–Wilk test, an analysis using ranks was performed. Differences between study products in the frequency of scores of 2 or greater (somewhat more or much more than usual) on the GI tolerability questionnaire were assessed using McNemar’s test.

Sensitivity analyses were performed to assess evidence of any condition by sequence interaction. Because no evidence was present for heterogeneity of response by sequence, the data from the two treatment sequences were pooled for analyses by study product.

## 3. Results

### 3.1. Study Subjects

**Figure 1:**
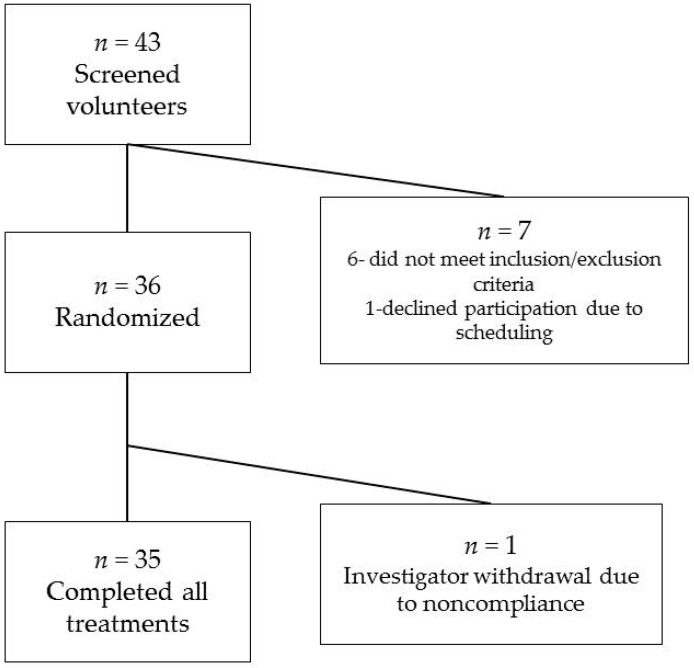
Subject flow through study.

Forty-three persons were screened for the study, but of those, six did not meet the inclusion/exclusion criteria and one met the screening criteria but was later screen failed because of scheduling conflicts with visits 2 and 3. A total of 36 subjects were randomized to a treatment sequence. One subject was terminated from the study before its completion because of inadequate compliance with study instructions. There were 32 subjects included in the glucose and insulin analyses because of missing samples from at least one of the test visits; 35 subjects were included in the satiety VAS, GI tolerability, and palatability analyses. Subject flow through the study is shown in Figure 1.

Study subject demographics are reported in Table 2. The subjects were primarily female, with a mean age of 46.2 years and a mean body mass index of 26.1 kg/m^2^. Each subject was given a copy of his or her diet record for the 24 h prior to test 1 and asked to replicate intake for the same period prior to test 2. The prior 24-h diet record returned on the day of test 2 was reviewed and any material deviations were noted. None of the subjects had deviations sufficiently large to warrant exclusion from the per protocol analysis in the judgment of the study director.

### 3.2. Blood Glucose and Insulin

**Figure 2:**
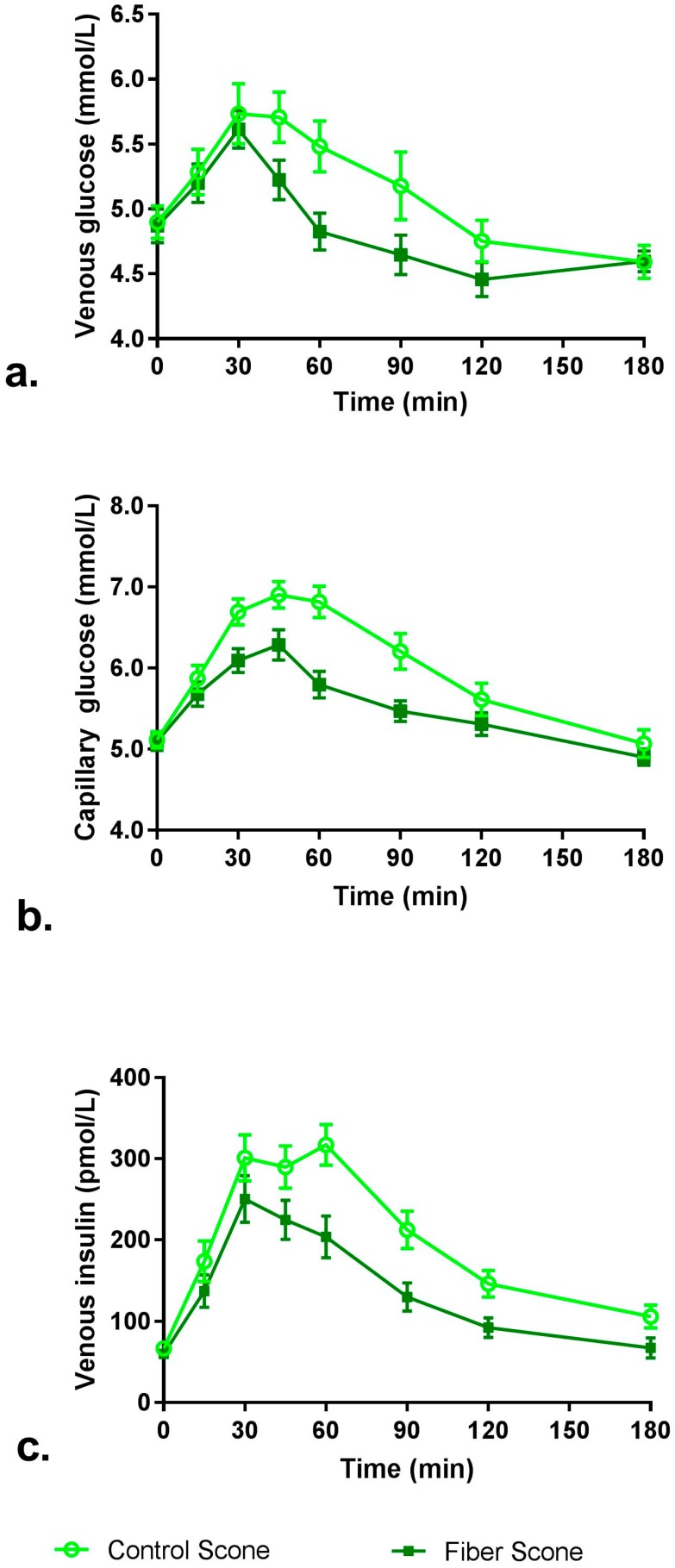
Mean post-prandial glucose and insulin concentrations over 180 min: (**a**) venous glucose; (**b**) capillary glucose (**c**) venous insulin. Error bars represent the standard error of the mean.

The time-course graph of venous and capillary glucose and venous insulin concentrations is presented in Figure 2, and iAUC and Cmax are shown in Table 3. Venous and capillary glucose iAUC_0–120 min_ and iAUC_0–180 min_ mean values were all 43–45% lower after consumption of the fiber scone compared to control scone (*p* < 0.05). Venous insulin iAUC_0–120 min_ and iAUC_0–180 min_ mean values were 35% and 40% lower, respectively, after consumption of the fiber scone compared to control scone (*p* < 0.05). Mean venous and capillary glucose and venous insulin Cmax levels were also significantly lower after consumption of the fiber scone compared to control scone (8%, 10% and 22% lower, respectively; *p* < 0.05).

### 3.3. Satiety VAS Scores

Results for niAUC satiety VAS scores are shown in Table 4. Hunger and desire to eat niAUC_0–180 min_ mean values were significantly lower after consumption of the fiber scone compared to control scone (*p* < 0.05). Fullness and prospective consumption niAUC_0–180 min_ mean values did not differ significantly between study products.

### 3.4. GI Tolerability Questionnaire

Results for the analyses of the frequency of scores of 2 or greater (somewhat more than usual and much more than usual) on the components of the GI tolerability questionnaire are shown in Table 5. There were no significant differences between study products in the number of subjects with ratings of somewhat more than usual or much more than usual for nausea, bloating, GI rumblings, flatulence, abdominal pain, or diarrhea.

### 3.5. Study Product Palatability

Results for the analyses of study product palatability are shown in Table 6. There was no significant difference between study products for any of the characteristics evaluated on the palatability questionnaire including appearance, texture, flavor, acceptance, healthiness or nutritiousness of the fiber scone and control scones. There were also no significant differences between study products for subjects’ agreement with the statements “I recommend the scone to family and friends” and “I tolerated the scone well, with no complaints”.

## 4. Discussion

The present study demonstrates that replacing a portion of refined wheat flour in a food with RS4 can reduce postprandial glucose and insulin concentrations and increase selected measures of satiety. The blood glucose and insulin findings align with results from a previous study administering the same RS4 in a muffin top to healthy adults [19]. In the present study, the percent reductions in iAUC_0–120 min_ glucose (venous and capillary) and iAUC_0–120 min_ insulin were greater than in the previously published study, which may be attributed to the higher dietary fiber content of the RS4-containing scone in the present study. Both study foods replaced a portion of refined wheat flour with VERSAFIBE 2470 resistant starch. This replacement resulted in an increase in TDF content and a decrease in available carbohydrate content which contributed to the reduced glucose and insulin responses. The absolute blood glucose values typically differ when measured intravenously or through capillary sampling, with capillary sampling typically displaying lower variation [25]. It is expected that the venous values will be a bit lower than the capillary values because capillary blood will have a value intermediate between that of venous and arterial blood. The present data from individual time points align with this previous observation.

The intended application of the novel RS4 in the present study is to replace digestible starch (e.g., wheat flour) in food products. Accordingly, the study was designed to provide data on the glycemic and insulinemic responses to food products prepared with non-resistant starch (control) or RS4 resistant starch. Therefore, the trial was designed in a manner that matched total carbohydrate content and portion size rather than available carbohydrate content. This deliberate choice in study design provides information relevant to the intended application of the ingredient studied (RS4). Glycemic response, which was evaluated in the present study, is often confused with glycemic index, which requires that the control food and test food be matched for 50 g available carbohydrate. These two concepts have been discussed in detail elsewhere [26].

Other types of RS4 have also been effective in lowering blood glucose compared to a control treatment. Four acute studies of RS4 containing distarch phosphate or phosphated distarch phosphate demonstrated reduction in postprandial blood glucose when administered in a beverage [8,14], a bar [9], and a cookie [16]. An acute study of maize hydroxypropyl distarch phosphate RS4 (38 g) in pancakes administered to healthy adults reported a significant reduction in postprandial glucose and insulin compared to control (0–180 min) [17]. A similarly designed study using the same RS4 (40 g) in pancakes reported no difference in blood glucose or insulin values over 180 min [18]. It should be noted that the RS4-containing pancake in both studies provided 0 g fiber. The inconsistency in blood glucose response to different RS4s emphasizes the need for evaluating health effects from consuming specific types of RS4, especially as new RS4s are developed.

Three studies examined the effect of a different type of RS4 on acute satiety response [11,14,18], and each study examined a different type of RS4. Satiety ratings in response to wheat phosphated distarch phosphate in a bar (10 g) [11] and potato distarch phosphate in a beverage (30 g) [14], and maize hydroxypropyl distarch phosphate in pancakes (40 g) [18], did not differ from those in control treatments. In contrast, the present study demonstrated that acid-hydrolyzed and heat-treated RS4 reduced hunger and desire to eat during the 180 min following consumption. The mechanism of action for this short-term effect is unclear and warrants further research. Additionally, long-term studies of RS4 on energy intake and weight management are needed to understand the full range of responses to consumption of this fiber.

Effects of long-term intake of wheat-based RS4 (phosphated distarch phosphate) were examined in two studies and published in three articles [10,12,13]. Martinez et al. (2010) compared the effects of high amylose maize resistant starch (RS2) and wheat phosphated distarch phosphate (RS4) on the gut microbiota [10]. While both types of RS impacted the gut microbiota, their effects were distinct, which reinforces the hypothesis that different types of RS may yield different health effects. A study in the Hutterite community, which has a high prevalence of metabolic syndrome, demonstrated that inclusion of RS4 did not affect measures of glycemic health, but improved blood lipids, waist circumference, and body composition [12]. The amount of RS4 or fiber consumed was not reported, which limits the ability to draw conclusions on long-term intake. An analysis of a subsample from the aforementioned study reported changes in gut microbiota composition and increased fecal short-chain fatty acid concentrations, suggesting that the gut microbiota may be a mediator for the changes in metabolic biomarkers [13]. This paper reported mean nutrient intake at baseline and after the two intervention periods. However, this was a subsample of the previous study and it is unclear whether the dietary data applies to the larger group in the previously published paper.

The glycemic response findings in the present study are supported by results from many previous studies of RS4. This study demonstrates that a reduction in post-prandial blood glucose and insulin can be achieved using an RS4 that is created through a novel chemical modification (acid hydrolyzed and heat-treated, instead of phosphate cross-linked). This is the first study to report significantly increased satiety (hunger, desire to eat) after consuming RS4. There are a few limitations of this study: (1) the study was conducted in healthy individuals, so we cannot draw conclusions related to chronic disease risk or management of existing conditions such as diabetes mellitus; and (2) the study foods (low fiber scone and high fiber scone) may not be culturally relevant to all countries and, thus, additional research is needed to evaluate glycemic and satiety response to culturally appropriate foods. Future research should examine the long-term effects of different RS4s to better understand the range of health effects from these ingredients.

## 5. Conclusions

In conclusion, this study demonstrated that a baked good, fortified with RS4 (acid hydrolyzed and heat treated maize starch) that replaced available carbohydrate from wheat flour, reduced postprandial plasma glucose response (venous and insulin) and correspondingly reduced plasma insulin. The study foods were administered in a practical portion size, emulating what consumers might find in the marketplace. This is the first study to report changes in two measures of satiety, hunger and desire to eat, after consuming RS4. The high fiber scone was well-tolerated and did not change gastrointestinal symptoms. Resistant starch type 4 has the potential for helping to fill the fiber gap in the Western diets while improving glycemic health.

## Figures and Tables

**Table 1:** Nutrient composition of study scones.

Per Serving, As-Eaten	Control Scone	Fiber Scone
Weight (g)	83.9	84.1
Calories (kcal)	328	270
Fat (g)	16.0	14.4
Saturated fat (g)	5.0	4.7
Protein (g)	7.1	6.1
Total Carbohydrates (g)	42.8	46.4
Available Carbohydrates (g)	38.8	28.9
Dietary Fiber (g) *	4.0	17.5
Sugars (g)	14.8	14.9

* VERSAFIBE^TM^ 2470 resistant starch provided 16.5 g dietary fiber in the Fiber Scone.

**Table 2:** Subject demographics.

Variable	All Participants (*n* = 35) ^1^
Age (y)	46.2 ± 2.2
Sex (male/female)	12/23
Race (white/nonwhite)	22/13
Ethnicity (non-Hispanic/Hispanic)	26/9
Weight (kg)	73.9 ± 2.1
Body mass index (kg/m^2^)	26.1 ± 0.5
Fasting capillary glucose (mmol/L)	5.04 ± 0.09

^1^ Values for continuous variables are mean ± standard error of the mean; values for categorical values are frequencies.

**Table 3:** Post-prandial glucose and insulin iAUC and Cmax (*n* = 32) ^1,2^.

Parameter	Control Scone	Fiber Scone	*p*-Value ^3^
Venous blood glucose			
iAUC_0–120 min_ (min × mmol/L)	69.65 ± 9.05	38.41 ± 5.77	0.0014
iAUC_0–180 min_ (min × mmol/L) ^4^	84.75 ± 11.43	48.29 ± 9.55	0.0039
Cmax (mmol/L) ^4^	6.44 ± 0.18	5.88 ± 0.12	0.0039
Capillary blood glucose			
iAUC_0–120 min_ (min × mmol/L) ^4^	139.14 ± 14.60	79.75 ± 8.10	0.0004
iAUC_0–180 min_ (min × mmol/L) ^4^	171.88 ± 19.65	94.63 ± 10.66	0.0003
Cmax (mmol/L) ^4^	7.49 ± 0.19	6.72 ± 0.16	0.0002
Venous insulin			
iAUC_0–120 min_ (min × pmol/L)	19,229 ± 1865	12,592 ± 1686	0.0005
iAUC_0–180 min_ (min × pmol/L)	23,850 ± 2138	14,192 ± 1901	<0.0001
Cmax (pmol/L) ^4^	392 ± 28	305 ± 26	0.0008

^1^ iAUC = incremental area under the curve, Cmax = maximum concentration. ^2^ Values are mean ± standard error of the mean. ^3^ *p*-values derived from repeated measure analysis of variance (ANOVA) with subject included as a random effect. ^4^ Values were ranked prior to ANOVA.

**Table 4:** Satiety visual analog scores (*n* = 35) ^1,2^.

Parameter	Control Scone	Fiber Scone	*p*-Value ^3^
Hunger niAUC_0–180 min_ (mm × min)	−1173 ± 896	−2840 ± 780	0.0316
Fullness niAUC_0–180 min_ (mm × min)	3926 ± 753	4028 ± 898	0.8858
Desire to eat niAUC_0–180 min_ (mm × min)	−1184 ± 918	−3046 ± 773	0.0135
Prospective consumption niAUC_0–180 min_ (mm × min)	−2098 ± 799	−2847 ± 672	0.1619

^1^ mm = millimeters, min = minutes, niAUC = net incremental area under the curve; ^2^ Values are mean ± standard error of the mean; ^3^ *p*-values derived from repeated measures analysis of variance with subject included as a random effect.

**Table 5:** Gastrointestinal Tolerability: Frequency of scores ≥ 2 (*n* = 35) ^1,2,3^.

Parameter	Control Scone	Fiber Scone	*p*-Value ^4^
Nausea	0 (0) ^3^	0 (0)	1.000
Bloating	1 (2.9)	0 (0)	1.000
GI Rumblings ^4^	4 (11.4)	1 (2.9)	0.2482
Flatulence	1 (2.9)	0 (0.0)	1.000
Abdominal Pain	0 (0.0)	0 (0.0)	1.000
Diarrhea	0 (0.0)	0 (0.0)	1.000

^1^ GI = gastrointestinal; ^2^ Scoring system: 0 = none, 1 = no more than usual, 2 = somewhat more than usual, 3 = much more than usual; ^3^ Values are *n* (%); ^4^ *p*-value generated from McNemar’s test for frequencies of values ≥ 2.

**Table 6:** Palatability Evaluation (*n* = 35) ^5^.

Parameter	Control Scone	Fiber Scone	*p*-Value ^6^
Appearance ^1^	7.74 ± 0.32	7.63 ± 0.32	0.4875
Texture ^1^	6.97 ± 0.34	6.40 ± 0.36	0.0743
Flavor ^1^	7.11 ± 0.04	6.69 ± 0.43	0.1185 ^7^
Acceptance ^1^	7.17 ± 0.36	6.77 ± 0.43	0.2271 ^7^
Healthy ^2^	5.91 ± 0.46	5.71 ± 0.44	0.4205
Nutritious ^3^	5.57 ± 0.44	5.23 ± 0.44	0.2092 ^7^
Recommend ^4^	0.17 ± 0.20	0.09 ± 0.21	0.5712
Tolerated ^4^	0.89 ± 0.20	0.97 ± 0.16	0.8601 ^7^

^1^ Scoring system: 1 (dislike extremely) to 10 (like extremely) on the appearance, texture, flavor, and overall acceptance of the scones; ^2^ Scoring system: 1 (not at all healthy) to 10 (extremely healthy); ^3^ Scoring system: 1 (very little) to 10 (very nutritious). ^4^ “I recommend the scone to family and friends” and “I tolerated the scone well, with no complaints” by marking rating of −2 (strongly disagree), −1 (disagree), 0 (no opinion), +1 (agree), or +2 (strongly agree); ^5^ Values are mean ± standard error of the mean; ^6^ *p*-value derived from repeated measures analysis of variance (ANOVA) that included subject as a random effect; ^7^ Individual values were ranked prior to ANOVA.

## Supplementary Materials

The following are available online at http://www.mdpi.com/2072-6643/10/2/129/s1, Table S1: Scone Formulations.
