# Supplementary material for: Type-4 Resistant Starch in Substitution for Available Carbohydrate Reduces Postprandial Glycemic Response and Hunger in Acute, Randomized, Double-Blind, Controlled Study
Source: Nutrients. 2018 Jan 26;10(2):129. doi: 10.3390/nu10020129 (PMC5852705; doi:10.3390/nu10020129)
Supplement: Supplementary file 1 [file nutrients-10-00129-s001.pdf]

Supplementary Table S1. Scone Formulations

Ingredient	Control Scone	Fiber Scone
VERSAFIBE 2470 Resistant starch [1]	0.0%	26.6%
Sugar, granulated	9.6%	9.6%
Baking powder	1.2%	1.2%
Baking soda	0.3%	0.3%
Non-iodized salt	0.2%	0.2%
Buttermilk powder	3.1%	3.1%
Citrus and vanilla flavoring	0.5%	0.5%
Cinnamon	0.2%	0.2%
Flour, high gluten	25.1%	8.1%
Flour, pastry	14.3%	2.6%
Vital wheat gluten	0.0%	3.0%
Cinnamon chips	5.8%	5.8%
Shortening, all purpose	13.4%	12.4%
Water	10.6%	10.6%
Whole eggs, frozen	15.7%	15.7%

[1] VERSAFIBE 2470 Resistant starch is manufactured by Ingredion Incorporated, Bridgewater, NJ 08807, USA
